# Updated prevalence rates of overweight and obesity in 11- to 17-year-old adolescents in Germany. Results from the telephone-based KiGGS Wave 1 after correction for bias in self-reports

**DOI:** 10.1186/s12889-015-2467-x

**Published:** 2015-11-06

**Authors:** Anna-Kristin Brettschneidera, Angelika Schaffrath Rosario, Ronny Kuhnert, Steffen Schmidt, Susanna Wiegand, Ute Ellert, Bärbel-Maria Kurth

**Affiliations:** Department of Epidemiology and Health Monitoring, Robert Koch Institute, General-Pape-Str. 62-66, 12101 Berlin, Germany; Department of Sports and Sports Science, Karlsruhe Institute of Technology, Engler-Bunte-Ring 15, 76131 Karlsruhe, Germany; Department of Pediatric Endocrinology and Diabetology, Charité Universitätsmedizin Berlin, Augustenburger Platz 1, 13353 Berlin, Germany

**Keywords:** Overweight, Obesity, Prevalence, Adolescents, KiGGS Wave 1

## Abstract

**Background:**

The nationwide “German Health Interview and Examination Survey for Children and Adolescents” (KiGGS), conducted in 2003–2006, showed an increase in the prevalence rates of overweight and obesity compared to the early 1990s, indicating the need for regularly monitoring. Recently, a follow-up—KiGGS Wave 1 (2009–2012)—was carried out as a telephone-based survey, providing self-reported height and weight. Since self-reports lead to a bias in prevalence rates of weight status, a correction is needed. The aim of the present study is to obtain updated prevalence rates for overweight and obesity for 11- to 17-year olds living in Germany after correction for bias in self-reports.

**Methods:**

In KiGGS Wave 1, self-reported height and weight were collected from 4948 adolescents during a telephone interview. Participants were also asked about their body perception. From a subsample of KiGGS Wave 1 participants, measurements for height and weight were collected in a physical examination. In order to correct prevalence rates derived from self-reports, weight status categories based on self-reported and measured height and weight were used to estimate a correction formula according to an established procedure under consideration of body perception. The correction procedure was applied and corrected rates were estimated.

**Results:**

The corrected prevalence of overweight, including obesity, derived from KiGGS Wave 1, showed that the rate has not further increased compared to the KiGGS baseline survey (18.9 % vs. 18.8 % based on the German reference).

**Conclusion:**

The rates of overweight still remain at a high level. The results of KiGGS Wave 1 emphasise the significance of this health issue and the need for prevention of overweight and obesity in children and adolescents.

## Background

The “German Health Interview and Examination Survey for Children and Adolescents” (KiGGS), which is part of the health monitoring system of the Robert Koch Institute, regularly collects health data from a nationwide, representative sample of children and adolescents. In the KiGGS baseline survey, which was an examination survey carried out in 2003–2006, height and weight of the participants were measured [1]. It was found that the prevalence rate of overweight, including obesity, in children and adolescents aged 3 to 17 years, had risen by about 50 %, and obesity had doubled compared to the early 1990s. The prevalence of overweight, including obesity, in adolescents aged 11 to 17 years had almost doubled, and the prevalence of obesity had nearly tripled [2].

Currently, a stabilisation of the prevalence rates of overweight and obesity in children and adolescents in developed countries has been reported [3], while the rates are still increasing in developing countries [4]. In Germany between 2004 and 2008, a significant downward trend for younger boys and girls between 4 to 7 years of age could be seen in one study, whereas for older children and adolescents aged 8 to 16 years, a plateau seems to have been reached [5]. The compulsory school enrolment examination showed a stagnation, or even a decrease, in the prevalence of overweight and obese children at the age of school entry [6].

However, the rates remain at a high level and still represent a significant health issue that requires regular monitoring. Recently, a follow-up of the KiGGS study—KiGGS Wave 1 (2009–2012)—was carried out as a telephone-based survey, providing self-reported height and weight from 11- to 17-year-old adolescents [7]. Analyses of self-reported height and weight data can underestimate the prevalence rates of overweight and obesity [8–11]. For the KiGGS baseline, it was shown that underreporting is stronger in girls than in boys, and is also stronger in overweight/obese individuals compared to normal-weight adolescents. Body perception emerged as a main predictor of the bias in self-reported height and weight, next to gender and weight status [8, 10, 12]. Therefore, either an individual correction of self-reported height and weight in order to determine the weight status or a correction of the prevalence rates of weight status derived from self-reports is necessary. Some of the authors developed and validated different approaches of correction based on self-reported and measured data of the KiGGS baseline survey [10, 13]. The correction procedure which directly corrects prevalence rates derived from self-reports by considering body perception [10], showed the smallest deviation from prevalence rates derived from measured data [13]. Therefore, this procedure [10] was replicated in the present paper in order to derive a new correction formula for KiGGS Wave 1, since the patterns of underreporting might vary over time. It has also been applied or replicated, respectively in other German studies in order to get improved prevalence estimates [14, 15].

A subsample of the KiGGS Wave 1 participants took part in the so-called ‘Motorik Modul’ (MoMo) focusing on motor fitness. For those participants, measurements of height and weight were collected [16]. The measurements from this small subsample provided the opportunity to improve the estimated prevalence rates of overweight and obesity derived from self-reported values of the representative KiGGS Wave 1 sample.

The aim of the present study was to obtain updated prevalence rates for overweight and obesity in 11- to 17-year olds living in Germany by applying the correction method described in Kurth and Ellert’s research [10].

## Methods

### Study population

The analyses presented are based on the data from KiGGS Wave 1. The goals, concept and design of KiGGS have been described elsewhere [1, 7, 17]. KiGGS Wave 1 (2009–2012), the follow-up of the KiGGS baseline study (2003–2006), was carried out as a telephone-based survey. An essential aim of KiGGS is to regularly provide population-based cross-sectional data on the health situation of children and adolescents aged 0–17 years living in Germany. Amongst others, the study population of KiGGS Wave 1 consists of re-invited participants from the baseline study (KiGGS cohort). A total of 5258 re-invited children and adolescents aged 11 to 17 years participated in KiGGS Wave 1 (response 73.9 %). The net sample was compared with the resident German population regarding particular population characteristics and an analysis of the relationship between the re-participation rate and certain characteristics collected in the baseline study, suggesting a mostly unbiased sample after taking the sample weights into account [7].

Trained study staff conducted standardised telephone interviews with adolescents aged 11 or older. For further information (e.g. socioeconomic status) and to collect information about the younger participants, parents of children and adolescents aged 0–17 years were interviewed. The software product Voxco Version 5.4.4.5 (Voxco Inc., Montréal QC, Canada) was used to manage the calls and collect the data. A written informed consent from parents or caregivers was required prior to the interviews. The Federal Office for Data Protection and the ethics committee of the Charité Medical University Berlin approved the survey.

Additionally, the ‘Motorik Modul’ (MoMo), which collected data on motor fitness and physical activity of children and adolescents aged 4 to 17 years in a physical examination, was part of KiGGS Wave 1 [16]. At the end of the telephone interview, approximately half of the KiGGS Wave 1 subjects were asked to participate in MoMo. If they gave their consent, they received information material and were contacted to make an appointment for the physical examination.

### Self-reports and anthropometric measurements

In the telephone interview, adolescents were asked to report their height (without shoes) and weight (without clothes) to an accuracy of 1 cm or 1 kg, respectively.

Trained staff, in the physical examination of the ‘Motorik Modul’ , took anthropometric measurements. Body height was measured without shoes to an accuracy of 0.1 cm using a portable stadiometer. Body weight, while the participant was wearing just underwear, was measured to the nearest 0.1 kg using a calibrated electronic scale.

Body mass index (BMI) in kg/m^2^ was calculated both from self-reported and from measured data. Weight status was determined using age- and gender-specific cut-offs for strong underweight (<3rd percentile), underweight (≥3rd percentile to <10th percentile), normal weight (≥10th percentile to ≤90th percentile), overweight (>90th percentile to <97th percentile) and obese (≥97th percentile), based on the national German reference [18].

### Body perception

Each adolescent’s body perception (BP) was examined by asking the following questions in the telephone interview: ‘Do you think you are …’ ‘much too thin’ , ‘a bit too thin’ , ‘exactly the right weight’ , ‘a bit too fat’ , or ‘much too fat’? [19]. Responses were classified into the following categories: (1) ‘too thin’ (summarising ‘much too thin’ and ‘a bit too thin’), (2) ‘right weight’ , and (3) ‘too fat’ (summarising ‘a bit too fat’ and ‘much too fat’).

### Socioeconomic status

The socioeconomic status of the participants was assessed by a multidimensional index score. The parents were asked to report their education and occupational qualifications, occupational status, and net income. This information was used to calculate the socioeconomic sum score, which was categorised into the following groups: (1) low, (2) moderate, and (3) high socioeconomic status [20].

### Statistical analysis/correction procedure

The analyses focused on 2509 boys and 2446 girls, aged 11 to 17 years, who were interviewed in KiGGS Wave 1. Cases with missing values for body perception (seven cases) were excluded, which led to a total sample size of 4948 adolescents (2505 boys and 2443 girls).

Measured height and weight from MoMo were available from 899 boys and 872 girls aged 11 to 17 years at the time of the KiGGS Wave 1 telephone interview. Due to the time lag between KiGGS Wave 1 and MoMo, there were 36 cases that were 17 years old at the time of the KiGGS Wave 1 telephone interview, but turned 18 when they participated in MoMo. These cases were included in the correction procedure. MoMo participants who did not give information about their body perception in the telephone interview were excluded from the correction procedure. Participants with a time lag between the telephone interview and the examination part of MoMo of greater than 3 months (90 days) were also excluded from the analyses. For the correction procedure of prevalence rates derived from self-reports of KiGGS Wave 1, weight status derived from height and weight measured in MoMo was thus available for 826 boys and 814 girls.

In previous analyses with data from KiGGS baseline, which provide both self-reported and measured values, correction of prevalence rates for weight status derived from self-reports with Kurth and Ellert’s [10] formula showed satisfying results [13]. Therefore, the correction procedure developed by Kurth and Ellert with data of KiGGS baseline (formula 16) [10] (i.e. the statistical procedure of directly correcting the prevalence rates) was replicated here in order to derive a new correction formula for KiGGS Wave 1. The derivation of a new correction formula was necessary since the amount and patterns of underreporting might vary (1) over time; and (2) with the change of survey mode (face-to-face interview in KiGGS baseline vs. telephone interview in KiGGS Wave 1). For the correction procedure, the prevalence based on measured height and weight from MoMo was used, as well as the information regarding individual body perception.

Let *R*_*k*_, *k* = 1…3 denote the prevalence of body perception (BP) category *k* in KiGGS Wave 1, i.e. *R*_*k*_ *= P (BP = k), k = 1…3* (‘too thin’ , ‘right weight’ , ‘too fat’). *Q*_*jk*_ is the uncorrected prevalence, based on self-reported height and weight in KiGGS Wave 1, of weight status category *j* in the group of adolescents with body perception *k*, i.e. it is the conditional probability

*Q*_*jk*_ = *P* (*BMI*_*reported*_ ∈ *I*_*j*_ | *BP* = *k*) (*j* = *1* … *6*; *k* = *1* … *3*), with

*I*_*1*_ *= Extremely underweight (<P3)*

*I*_*2*_ *= Underweight (≥P3 - < P10)*

*I*_*3*_ *= Normal Weight (≥P10 - < P90)*

*I*_*4*_ *= Overweight (≥P90 - < P97)*

*I*_*5*_ *= Obese (≥P97)*

*I*_*6*_ *= Self-reported height and/or weight missing*

Here, category *I*_6_ was newly introduced to represent adolescents with missing values for weight status derived from self-reports, but with available information on weight status derived from measurements and body perception.

The corrected prevalence rate for weight status category *j* in KiGGS Wave 1, *P *(ΒΜΙ_*corrected*_ ∈ Ι_*i*_), is then given by:

I $$ P\left(\mathrm{B}\mathrm{M}{\mathrm{I}}_{corrected}\in {\mathrm{I}}_i\right)={\displaystyle \sum_{k=1}^3\left[{\displaystyle \sum_{j=1}^6{\alpha}_{{}_{ijk}}{Q}_{jk}}\right]}{R}_k\left(i = 1\dots 5\right), $$

with *I*_1_ … *I*_5_ defined as above, where the relationship between measured and self-reported values is captured by the factors ∝_*ijk*_, which are the conditional probabilities for measured weight status category *i* in the group of adolescents with body perception *k* and weight status category *j* based on self-reported height and weight, i.e. ∝ _*ijk*_ = *P* (*BMI*_*measured*_ ∈ *I*_*i*_ | *BMI*_*reported*_ ∈ *I*_*j*_ , *BP* = *k*), with *I*_1_ … *I*_6_ defined as above.

The factors ∝_*ijk*_ were determined in the MoMo subsample. Weight status based on measured values was taken from MoMo, whereas information about weight status based on self-reports and body perception was taken from the telephone interview in KiGGS Wave 1. Then, 95 % confidence intervals for the corrected prevalence rates were estimated via a bootstrap procedure with 2000 replicates by resampling 166 sample points with replacement from the 167 original sample points [21] and using the 2.5 and 97.5 % quantiles of the bootstrapped distribution as the confidence limits. These confidence intervals include the effect of the additional uncertainty introduced by the correction procedure.

All analyses, including the correction procedure, were performed with the survey procedures of SAS release 9.4 (SAS Institute Inc., Cary, NC, USA, 2012), taking sample weights and the clustering in sample points into account. The analyses were both conducted separately for boys and girls and for the total sample. The sample weights were used to correct for possible deviations of the sample from the population structure (as of December 31, 2010) with regard to age, gender, region, parental education, and nationality (whether or not they were German). Furthermore, they included a correction for selective dropout based on a logistic regression modelling of the reparticipation probability [7].

For a comparison of the corrected prevalence rates derived from KiGGS Wave 1 with the prevalence rates from the KiGGS baseline study, the latter ones were recalculated using sample weights analogous to KiGGS Wave 1 (i.e. using the population structure as of December 31, 2010 and including parental education in the calculation of the sample weights) [7].

## Results

Table 1 illustrates the characteristics for both the study population of KiGGS Wave 1 with data derived from the telephone interview and for the subsample of KiGGS Wave 1 that participated in the MoMo physical examination. Prevalence rates derived from self-reported height and weight in the KiGGS sample were 7.8 % for overweight and 4.0 % for obesity. The rates based on self-reports from the MoMo subsample were similar (overweight: 8.0 %; obesity: 3.8 %). The conditional probabilities ∝_*ijk*_ required for the correction procedure are displayed in Table 2. Using ∝_*ijk*_ and *Q*_*jk*_ from Table 2 and *R*_*k*_ from Table 1, the corrected prevalence rates of weight status were estimated for KiGGS Wave 1 using formula (I).

**Table 1:** Description of the study population of KiGGS Wave 1 and the subsample of MoMo

	KiGGS wave 1						MoMo subsample					
	All (*n* = 4 948)		Boys (*n* = 2 505)		Girls (*n* = 2 443)		All (*n* = 1 640)		Boys (*n* = 826)		Girls (*n* = 814)	
	*N* ^a^	%^b^	*N* ^a^	%^b^	*N* ^a^	%^b^	*N* ^a^	%^b^	*N* ^a^	%^b^	*N* ^a^	%^b^
Age												
11–13 years	2155	43.0	1108	43.4	1047	42.6	809	47.0	405	45.0	404	49.2
14–17 years	2793	57.0	1397	56.6	1396	57.4	831	53.0	421	55.0	410	50.8
Weight status derived from uncorrected self-reported height and weight (Q_i_)												
Extremely underweight	159	3.4	68	2.9	91	3.9	58	4.2	24	3.1	34	5.4
Underweight	324	6.5	141	5.7	183	7.3	107	6.4	45	5.4	62	7.6
Normal weight	3608	78.3	1826	78.8	1782	77.9	1223	77.5	623	77.8	600	77.2
Overweight	292	7.8	165	7.8	127	7.7	88	8.0	49	8.5	39	7.5
Obese	152	4.0	94	4.8	58	3.2	41	3.8	25	5.2	16	2.3
Missing value	413		211		202		123		60		63	
Body perception (R_k_)												
Too thin	732	14.9	468	19.1	264	10.5	255	15.5	157	17.7	98	13.1
Right weight	2920	56.3	1438	55.2	1482	57.3	1002	57.3	504	58.9	498	55.5
Too fat	1296	28.9	599	25.6	697	32.2	383	27.3	165	23.4	218	31.4
Socio-economic status												
Low	590	22.6	316	24.1	274	20.9	159	22.7	81	23.3	78	21.9
Moderate	3118	59.7	1552	57.8	1566	61.7	1069	60.7	532	59.8	537	61.7
High	1178	17.7	608	18.0	570	17.3	411	16.6	212	16.8	199	16.4
Missing value	62		29		33		1		1			

^a^Unweighted

^b^Weighted

**Table 2:** Weight status categories derived from measured height and weight according to weight status categories derived from reported values and according to body perception

	All							Boys							Girls						
∝_*ijk*_	BMI_*reported*_						*Q*_*jk*_	BMI_*reported*_						*Q*_*jk*_	BMI_*reported*_						*Q*_*jk*_
	I_1_	I_2_	I_3_	I_4_	I_5_	I_6_		I_1_	I_2_	I_3_	I_4_	I_5_	I_6_		I_1_	I_2_	I_3_	I_4_	I_5_	I_6_	
BMI_*measured*_								Body perception = 1 (too thin)													
I_1_	51.4 %	9.7 %	2.4 %			12.8 %	13.0 %	61.1 %	19.2 %	2.3 %			15.8 %	9.8 %	43.0 %	3.1 %	2.8 %			6.7 %	19.1 %
I_2_	21.7 %	49.9 %	11.0 %			14.5 %	19.5 %	31.8 %	40.5 %	14.3 %			8.5 %	17.2 %	13.1 %	56.5 %	4.9 %			26.7 %	23.7 %
I_3_	26.9 %	40.4 %	86.1 %			72.7 %	58.6 %	7.1 %	40.3 %	82.9 %			75.7 %	62.2 %	43.9 %	40.4 %	92.3 %			66.6 %	51.8 %
I_4_			0.4 %							0.6 %											
I_5_																					
I_6_	-	-	-	-	-	-	8.9 %	-	-	-	-	-	-	10.8 %	-	-	-	-	-	-	5.4 %
								Body perception = 2 (right weight)													
I_1_	11.9 %	1.5 %	0.1 %			0.9 %	1.9 %	30.9 %	4.3 %	0.2 %				1.3 %	5.9 %					1.9 %	2.6 %
I_2_	20.8 %	26.6 %	1.0 %			1.0 %	5.1 %	12.9 %	38.2 %	0.9 %				3.3 %	23.4 %	20.2 %	1.1 %			2.1 %	7.0 %
I_3_	63.5 %	69.9 %	95.3 %	40.7 %		86.8 %	82.7 %	56.2 %	57.6 %	93.8 %	57.4 %		78.7 %	84.2 %	65.8 %	76.6 %	96.9 %			95.7 %	81.3 %
I_4_	3.8 %		3.5 %	45.2 %	63.5 %	0.2 %	1.8 %			4.8 %	42.6 %	73.0 %		2.7 %	5.0 %		1.9 %	51.6 %		0.4 %	1.0 %
I_5_		2.0 %	0.2 %	14.0 %	36.5 %	11.1 %	0.2 %			0.3 %		27.1 %	21.3 %	0.3 %		3.2 %	0.1 %	48.4 %	100.0 %		
I_6_	-	-	-	-	-	-	8.2 %	-	-	-	-	-	-	8.3 %	-	-	-	-	-	-	8.1 %
								Body perception = 3 (too fat)													
I_1_							0.1 %							0.1 %							0.2 %
I_2_						1.7 %	0.4 %							0.2 %						2.6 %	0.5 %
I_3_		100.0 %	75.2 %	5.3 %		23.1 %	54.2 %		100.0 %	72.4 %	1.5 %		21.1 %	50.0 %			77.0 %	8.3 %		24.2 %	57.7 %
I_4_	100.0 %		18.4 %	49.3 %	3.2 %	19.7 %	20.8 %			23.2 %	65.1 %	5.0 %	9.0 %	21.8 %	100.0 %		15.2 %	36.8 %		25.6 %	19.9 %
I_5_			6.4 %	45.4 %	96.8 %	55.5 %	12.2 %			4.4 %	33.4 %	95.0 %	69.9 %	16.2 %			7.8 %	54.9 %	100.0 %	47.5 %	9.0 %
I_6_	-	-	-	-	-	-	12.3 %	-	-	-	-	-	-	11.7 %	-	-	-	-	-	-	12.8 %

*BMI*_*measured*_: derived from measured height and weight collected in MoMo

*BMI*_*reported*_: derived from self-reported height and weight collected in the telephone interview from KiGGS Wave 1 participants which also participated in MoMo

I_1_ … I_5_: i-th category for BMI and j-th category for BMI_reported_ (i.e., 1 = extremely underweight, 2 = underweight, 3 = normal weight, 4 = overweight, 5 = obese)

I_6_: Missing value for self-reported height and/or weight

*Q*_*jk*_ Prevalence rates of weight status categories j derived from self-reported height and weight according to body perception = k in KiGGS Wave 1

∝_*ijk*_ = *P* (*BMI*_*measured*_ ∈ *I*_*i*_ | *BMI*_*reported*_ ∈ *I*_*j*_ , *BP* = *k*)

**Fig. 1:**
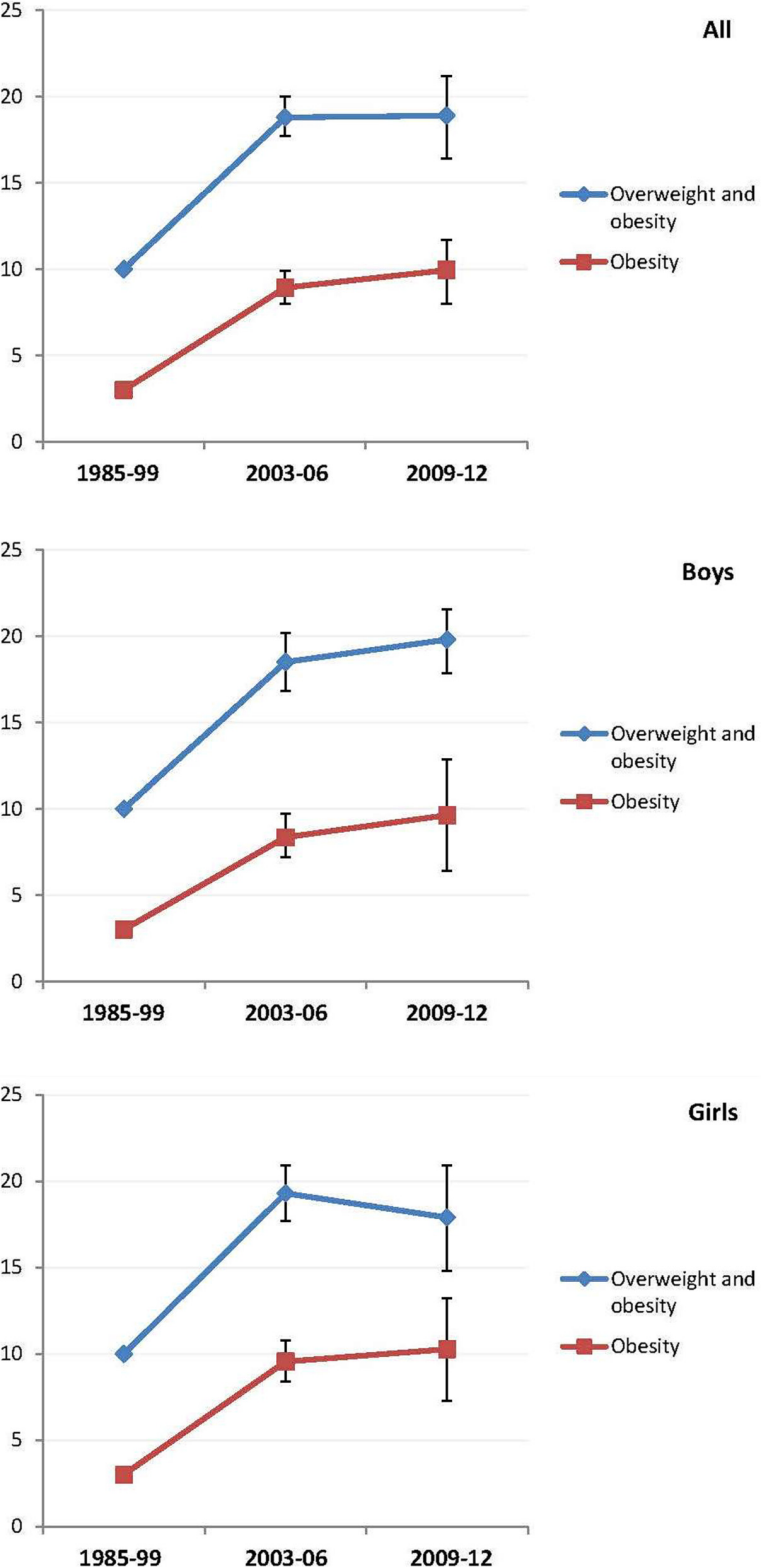
Comparison of prevalence rates of obesity and overweight, including obesity, for adolescents aged 11 to 17 years over time with data derived from Kromeyer-Hauschild (1985–99) [18], KiGGS baseline survey (2003–06) and KiGGS Wave 1 (2009–12)

Prevalence rates of weight status based on self-reported values and the corrected prevalence rates for KiGGS Wave 1 are presented in Table 3. The correction procedure led to remarkably higher prevalence estimates of overweight and obesity, especially in girls, which underlines the importance of using a correction. The prevalence of overweight, including obesity, has not further increased compared to the KiGGS baseline survey (KiGGS baseline: 18.8 % (95 % CI 17.7–20.0) vs. KiGGS Wave 1: 18.9 % (95 % CI 16.4–21.2)) (Fig. 1).

**Table 3:** Comparison of prevalence rates derived from measured (KiGGS baseline), self-reported (KiGGS Wave 1), and corrected (KiGGS Wave 1) data in 11- to 17-year old adolescents in Germany

Weight status	BMI^KiGGS baseline^_measured_		BMI^KiGGS1^_reported_		BMI^KiGGS1^_corrected_	
	%	95 % Cl	%	95 % Cl	%	95 % Cl
Boys	*n* = 3 477		*n* = 2 294		*n* = 2 505	
Extremely underweight	2.3	1.7–3.0	2.9	2.0–4.0	2.7	1.6–4.2
Underweight	5.4	4.5–6.4	5.9	4.7–7.3	5.0	3.2–6.9
Normal weight	73.9	72.0–75.7	78.7	75.9–81.3	72.4	68.5–76.4
Overweight	10.1	8.9–11.3	7.8	6.3–9.7	10.2	7.8–12.8
Obese	8.4	7.2–9.7	4.8	3.6–6.3	9.6	7.2–11.8
Girls	*n* = 3 302		*n* = 2 241		*n* = 2 443	
Extremely underweight	1.7	1.3–2.4	3.9	3.0–5.1	1.3	0.7–2.2
Underweight	4.8	4.1–5.6	7.3	6.0–8.8	3.9	2.6–5.5
Normal weight	74.2	72.3–76.0	77.9	75.4–80.3	76.8	73.2–80.2
Overweight	9.7	8.5–11.0	7.7	6.0–9.8	7.6	5.3–10.4
Obese	9.6	8.4–10.8	3.2	2.2–4.6	10.3	7.3–13.2
Total	*n* = 6 779		*n* = 4 535		*n* = 4 948	
Extremely underweight	2.0	1.6–2.5	3.4	2.7–4.2	1.9	1.3–2.7
Underweight	5.1	4.5–5.8	6.6	5.7–7.6	4.6	3.4–5.8
Normal weight	74.0	72.6–75.4	78.3	76.4–80.1	74.7	71.8–77.5
Overweight	9.9	9.1–10.8	7.7	6.6–9.0	8.9	7.0–10.9
Obese	8.9	8.0–9.9	4.0	3.2–5.0	10.0	8.0–11.7

After correction, 8.9 % (95 % CI 7.0–10.9) of the adolescents were overweight (boys: 10.2 % (95 % CI 7.8–12.8); girls: 7.6 % (95 % CI 5.3–10.4)) and 10.0 % (95 % CI 8.0–11.7) obese (boys: 9.6 % (95 % CI 7.2–11.8); girls: 10.3 % (95 % CI 7.3–13.2)). In comparison to prevalence from the KiGGS baseline survey (2003–2006), which was based on measured values, there is a slight, but not significant, decrease in the prevalence rate of overweight (KiGGS baseline: 9.9 % (95 % CI 9.1–10.8) vs. KiGGS Wave 1: 8.9 % (95 % CI 7.0–10.9)), whereas for obesity, a slight non-significant increase is seen (KiGGS baseline: 8.9 % (95 % CI 8.0–9.9) vs. KiGGS Wave 1: 10.0 % (95 % CI 8.0–11.7)). Gender differences are seen for overweight, but not for obesity. In girls, the prevalence of overweight decreased (n.s.), whereas in boys, it remained nearly unchanged (Table 3).

## Discussion

The corrected prevalence rates derived from KiGGS Wave 1 for overweight, including obesity, in adolescents aged 11 to 17 years in Germany was 18.9 % (boys: 19.8 %; girls: 17.9 %). In comparison to the prevalence from the KiGGS baseline survey (2003–2006) (total: 18.8 %), a stagnation in the prevalence of overweight, including obesity, has been reached. Thus the previously reported plateauing of the prevalence of overweight, including obesity, in adolescents living in Germany [5] can be confirmed by this current nationwide sample. However, there is still a slight tendency for increases (n.s.) in the obesity prevalence in both boys and girls. This indicates that in this age group, a higher percentage of those with overweight are obese compared to the rates from the KiGGS baseline survey, emphasising the significance of this health issue.

Similar results have been seen in the “German Health Interview and Examination Survey for Adults” (DEGS1), conducted in 2008–2011. DEGS1 showed, in comparison to studies from the 1990s, no further increase in the prevalence of overweight in women, whereas for men, a slight increase could be seen. However, the prevalence rates for obesity increased remarkably in both men and women, especially in young adults (aged 25 to 34 years) [22]. Similar trends have also been reported both for children and adults on an international level [23, 24]. The growing number of obese adolescents and young adults is alarming. Overweight and obese adolescents are more likely to stay obese into adulthood [25, 26] and when they themselves become parents and stay overweight or obese, their children are again more likely to be overweight or obese [27]. This cycle of the obesity epidemic needs to be interrupted since overweight and obesity is often accompanied by an increased risk for noncommunicable diseases like cardiovascular diseases, diabetes, cancer, and mental health problems [28–30]. This indicates a need for the prevention of overweight and obesity. Since the age of leaving kindergarten and starting primary school has been identified as a critical period for the development of overweight [31, 32], early childhood offers an effective starting point for preventive interventions. To avoid the increase in the prevalence rate of overweight after school entry, prevention should take place, at the latest, during the kindergarten years [31].

On a national level, the action plan “IN FORM - German national initiative to promote healthy diets and physical activity” was implemented with the aim of achieving lasting improvements with regard to the health behaviour of the population [33]. For 2016, a law for the enhancement of health promotion and prevention is planned by the Federal Ministry of Health [34]. The health goal “growing up healthy” (“gesund aufwachsen”) [35] is embedded in this law and gives hope for intensified actions.

This study has strengths and limitations. Strengths include the large sample size, the wide age range covered (11–17 years) and the fact that the study was based on a nationwide sample. A further strength is that a correction procedure was applied instead of the uncritical use of self-reported values. The comparison of corrected with uncorrected rates of KiGGS Wave 1 emphasises the need for a correction (e.g. after correction, the obesity rates in boys doubled and in girls, tripled). Due to the time lag and different interview modes between KiGGS baseline and KiGGS Wave 1, the method of the correction procedure Kurth and Ellert [10] developed was replicated with the self-reports derived from telephone-based KiGGS Wave 1 and the data of the measured values of a subsample. Furthermore, the existing correction procedure was extended to include missing values on self-reported height and/or weight, which occurred in 8 % of the study population, and confidence intervals were added to the corrected prevalence rates.

One important limitation of this study is the time lag between the telephone interview where self-reported data of height and weight were collected and the physical examination of MoMo, where measurements were obtained. For the derivation of the correction factors, the sample was restricted to participants with a time lag of less than 3 months. A further limitation could be that KiGGS Wave 1 was based on a longitudinal sample, thus the adolescents interviewed were part of the KiGGS cohort and were thus asked to participate in KiGGS for a second time. This could lead to a selection bias. However, the sample weights used in the analyses include a correction for the dropout probability. In the MoMo subsample, there might also be a selection bias that might even be stronger compared to the data of KiGGS Wave 1. Therefore, it was preferred to correct self-reports of KiGGS Wave 1 instead of using the measured values from the MoMo subsample. Still, there might be some selection bias left in the corrected prevalence rates, even after taking body perception and weight status based on self-reported values into account, so that the corrected prevalence rates for overweight and obesity might still be somewhat too low, but much less so than uncorrected rates. A further advantage of the larger sample size of KiGGS Wave 1 is increased stability of the estimated rates.

## Conclusion

In German adolescents aged 11 to 17 years, a plateau in the prevalence rate of overweight, including obesity, has been reached, but the rates still remain at a high level. For obesity, there is still a slight tendency for an increase (n.s.) in the prevalence seen. This emphasises the significance of this health issue and the need for prevention of overweight and obesity in children and adolescents.

## Abbreviations

BMI: Body mass index
DEGS1: German health interview and examination survey for adults
KiGGS: German health interview and examination survey for children and adolescents
MoMo: Motorik Modul
