# Erratum to: ‘Updated prevalence rates of overweight and obesity in 11- to 17-year-old adolescents in Germany. Results from the telephone-based KiGGS Wave 1 after correction for bias in self-reports’

**DOI:** 10.1186/s12889-016-2855-x

**Published:** 2016-03-09

**Authors:** Anna-Kristin Brettschneidera, Angelika Schaffrath Rosario, Ronny Kuhnert, Steffen Schmidt, Susanna Wiegand, Ute Ellert, Bärbel-Maria Kurth

**Affiliations:** Department of Epidemiology and Health Monitoring, Robert Koch Institute, General-Pape-Str. 62-66, 12101 Berlin, Germany; Department of Sports and Sports Science, Karlsruhe Institute of Technology, Engler-Bunte-Ring 15, 76131 Karlsruhe, Germany; Department of Pediatric Endocrinology and Diabetology, Charité Universitätsmedizin Berlin, Augustenburger Platz 1, 13353 Berlin, Germany

Unfortunately, the original version of this article [1] contained an error. The spelling of the author [Anna-Kristin Brettschneidera] name was incorrect. This has been corrected above and also included correctly below:

Anna-Kristin Brettschneider
